# Effects of BMI and grip strength on older adults' falls—A longitudinal study based on CHARLS

**DOI:** 10.3389/fpubh.2024.1415360

**Published:** 2024-12-04

**Authors:** Lei Huang, Xiaoxin Shen, Yuliang Zou, Yanming Wang

**Affiliations:** ^1^Department of Geriatrics, Wuhan Wuchang Hospital (Wuchang Hospital Affiliated to Wuhan University of Science and Technology), Wuhan, Hubei, China; ^2^Center of Health Management, Department of Global Health, School of Public Health, Wuhan University, Wuhan, China; ^3^Department of Infectious Diseases, Wuhan Wuchang Hospital (Wuchang Hospital Affiliated to Wuhan University of Science and Technology), Wuhan, Hubei, China

**Keywords:** older adults, body mass index (BMI), grip strength, gender difference, falls

## Abstract

**Background:**

Body Mass Index (BMI) and grip strength are potentially important risk factors for falls among the older adults. Currently, there is no consensus on the combined effects of grip strength and BMI on falls in the older adults, particularly among the Chinese older adults.

**Objective:**

To investigate the incidence of falls among older adults in China and explore the association between BMI and grip strength and the risk of falls in older adults.

**Methods:**

Data of participants over 60 in China Health and Retirement Longitudinal Study in 2011 and 2013 were collected. Sociodemographic variables, lifestyle, chronic disease status, history of falls and depression and cognitive status were obtained through the 2011 baseline questionnaire. Height, weight and grip strength were collected from a unified physical examination in 2011. Falls during the follow-up period were obtained through the follow-up questionnaire in 2013. Logistic regression was used to explore the association between BMI and grip strength and the risk of falls.

**Results:**

Three thousand six hundred and eighty-five participants over 60 (67.14 ± 6.08) were included, with a fall incidence rate of 17.37%. The fall incidence rate in females (21.15%) was higher than that in male (13.46%). After adjusting covariates, high grip strength was associated with lower falls risks in general population (OR = 0.76; 95 *CI*: 0.630–0.923) and males (OR = 0.68; 95 *CI*: 0.503–0.919). Underweight was associated with lower falls risks in general population (OR = 0.77; 95 *CI*: 0.595–0.981) and females (OR = 0.69; 95 *CI*: 0.486–0.962) compared to the normal BMI group. Compared with the low grip strength group, females with high grip strength (OR = 0.54; 95 *CI*: 0.29–0.98) had a lower risk of falls in the underweight BMI group and males with high grip strength (OR = 0.63; 95 *CI*: 0.43–0.92) had lower risk of falls in the normal BMI group.

**Conclusions:**

High grip strength and underweight BMI are independently correlated with a lower fall risk, which varies between males and females.

## 1 Background

Falls are a widespread public health concern worldwide, costing more than 30,000 disability adjusted life years (DALYs) each year, exceeding the sum of traffic injuries, drowning, burns, and poisoning (1). The older adults population becomes more prone to falls and has the highest risk of death or disability due to falls given the atrophy and degradation of sensory and cognitive functions, as well as the decrease in physical fitness. From 2010 to 2019, the incidence of falls and the disease burden amongst the older adults in Chinese Mainland have increased yearly. In 2019, 3,799.4 falls per 100,000 people were recorded, with a fall mortality rate of 39.2 per 100,000 people and DALYs for falls of 1,238.9 per 100,000 people (2). After a fall, the physical function of the older adults further declines, and the fear of falling intensifies, thereby restricting new subjective activities (3). This condition causes cognitive dissonance, falls, social vulnerability, depression, and weakened self-awareness (4). As a result, the quality of life of the older adults population is reduced and the risk of falling is further increased, triggering a new vicious circle (5, 6). Furthermore, falls can cause mental and financial stress to caregivers.

In addition to gender and age, the most important influencing factors of falls include previous fall histories, muscle strength, gait and balance disorders and the use of specific drugs. The number of chronic diseases, pain, physical weakness, cognitive impairment, depression, fear of falls are the risk factors of falls (7–13).

Thus far, there is no consensus on the impact of muscle strength on the risk of falls in the older adults. Some studies suggest that muscle strength and fitness levels are associated with the risk of fractures, and muscle strength and performance may reduce the risk of falls (14, 15). Hong et al. pointed out that after adjusting confounding factors, such as age and comorbidity index, lean body mass was associated with lower fracture risk, and higher muscle strength became a mediator of fall risk (16). However, the results were different in aged female. Dowling et al. used waist circumference indicators to measure the degree of obesity in the older adults, identifying the sex specificity of obesity, muscle strength and falls (17). The results showed that obesity and low muscle strength were independently associated with falls, whereas abdominal obesity and low muscle strength could only predict fall risk in aged male. However, studies also showed that muscle strength is not associated with the risk of falls and fractures (18). Research from PURE indicates that there is no significant association between falls and injuries (19). Further research is required to demonstrate whether muscle strength is a predictive factor for falls. In addition, numerous studies have shown that obesity is associated with an increased risk of falls in the older adults (20). Some studies have also found a U-shaped association between BMI and fall risk, where underweight and obesity possibly associated with higher fall risk (21).

Muscle strength and obesity cannot be viewed in isolation. In the older adults, aging is associated with a gradual increase in fat and a decrease in muscle mass. Obesity and muscle mass may interact and jointly affect the health status of the older adults population. Aged people with insufficient muscle mass and obesity may have a higher risk of disease. However, only few studies explore the correlation amongst muscle strength, obesity and fall risk presently, and the results are inconsistent. Whether the results can be extrapolated to the older adults population in China remains to be debated. And most studies mainly use waist circumference to measure obesity levels, which may not accurately measure the degree of obesity amongst participants given the differences in the distribution of body fat between males and females (22).

In the context of the deepening aging population in China, our study aims to explore the combined impact of BMI and grip strength on the risk of falls among Chinese older adults individuals. This research seeks to provide targeted recommendations for predicting and controlling the risk of falls in older adults.

## 2 Methods

### 2.1 Participants

Our study is based on data from two waves of the China Health and Retirement Longitudinal Study (CHARLS) in 2011 and 2013. The CHARLS collected a high-quality nationally representative sample of Chinese residents ages 45 and older. The baseline survey was conducted in 2011, covering 17,500 individuals in 150 counties/districts, 450 villages/resident committees (23). Full details about CHARLS can be found elsewhere (24).

**Figure 1:**
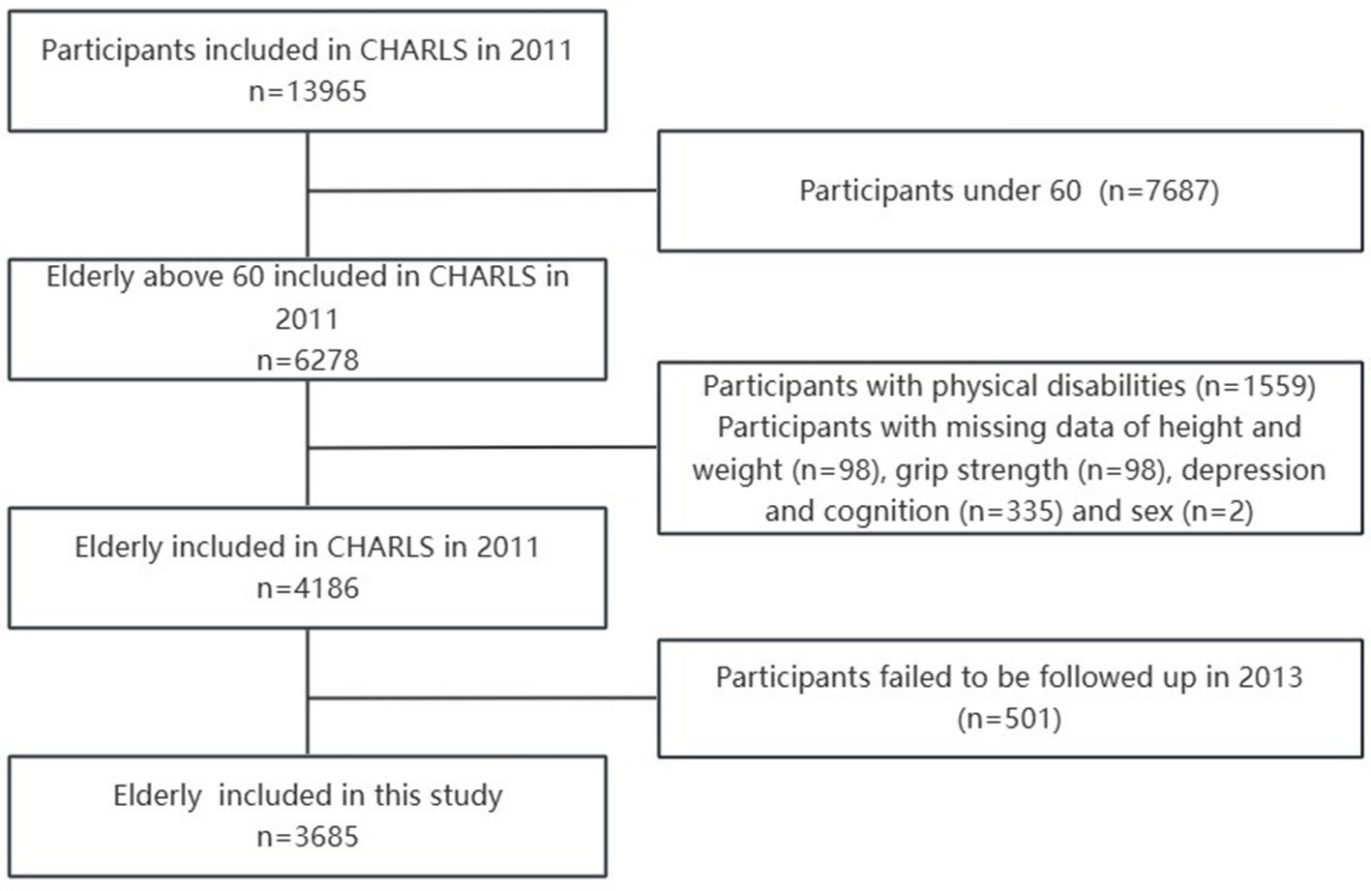
Flowchart of the study participants included in the study from 2011 to 2013.

In this study, we pooled, cleaned and filtered data from CHARLS in 2011 and 2013. After excluding participants with physical disabilities, as well as those without social demographic information and those who did not complete physical examinations, we included 4,186 participants over 60 at baseline. Three thousand six hundred and eighty-five participants completed follow-up surveys in 2013, with a follow-up rate of 88%. As shown in Figure 1.

### 2.2 BMI, grip strength, and follow-up falls

BMI was calculated based on the height (m) and weight (kg) measured in the baseline physical examination in 2011. Considering that the study population consists of older adults people in China with a relatively low proportion of obesity, we merged the overweight and obese groups. In this study, BMI is divided into three groups: underweight, normal weight, and overweight/obese according to Chinese Dietary Guidelines 2022 and Appropriate range of body mass index and body weight management guidelines for Chinese oldest old (25, 26). The cut-off values for different age groups are detailed in Supplementary Table S1.

Grip strength was measured at baseline with the WL-1000 grip strength tester. Grip strength was measured twice for each hand, with each hand taking turns to measure. The maximum value of the dominant hand measurements was obtained to represent the overall grip strength level. Based on The National Physical Fitness Testing Standard (revised in 2023) and Chinese Dietary Guidelines 2022, we grouped the grip strength of older adults according to different age groups. The cut-off values are shown in Supplementary Table S2 (26, 27).

In the 2013 follow-up questionnaire, the question “Have you fallen down in the last 2 years?” was used to identify falls that occurred between baseline and follow-up. The “past 2 years” refer to the period of 24 months from the date of the baseline to the date of the interview in 2013. Investigators verified the authenticity of participants' responses by seeking evidence from family members and reviewing medical records.

### 2.3 Depression and cognition

We used The Center for Epidemiological Studies Depression-10 (CES-D-10) to measure global depressive symptoms. Each item has four options on this scale, namely, “rarely or none of the time (< 1 d),” “some or a little of the time (1–2 d),” “occasionally or moderate amount of time (3–4 d),” “most or all the time (5–7 d).” The overall score ranged from 0 to 30, with higher scores and more pronounced depressive symptoms.

Cognition was tested by CHARLS Harmonized Cognitive Assessment Protocol (HCAP) (CHARLS-HCAP), which has been proven effective and feasible to apply to the older adults in China (28). Cognition is scored in three sections: calculating, drawing and memory function. The calculating section contains five items, asking the respondents the result of subtracting 7 from 100 in sequence. The participants are required to imitate a picture with overlapping pentagrams and redraw them. The memory function section consists of 15 items, of which five items assess the accuracy of older adults people's cognition of the year, month, day, day of the week and season. The 10 other items require participants to recall the 10 randomly selected words. The total score for the cognitive part is 21 points, and the higher score indicates better cognitive function.

### 2.4 Other covariates

We have adjusted the sociodemographic characteristics, lifestyles and health conditions related to falls as much as possible. Sociodemographic characteristics include age, gender, marital status (married with spouse present, married but not living with spouse temporarily, separated and divorced/widowed/never married) and education (illiterate, home school/elementary school, middle school, high school/vocational school bachelor degree or above). Lifestyles include smoking status (Yes or No now or in the past) and alcohol drinking status in the past year (more than once a month, less than once a month and no). We use the number of chronic diseases and the history of falls (zero, one, and two and above) to reflect the health condition of the older adults.

### 2.5 Statistical analysis

Continuous variables that satisfy normality are described by mean ± standard deviation (SD), those that do not satisfy normality are described by median (interquartile range), and categorical variables were represented by frequency and composition ratio *N* (%). The chi square test was used to compare the distribution of categorical variables between fall and non-fall groups, and the *Z*-test was used for pairwise comparisons between multiple groups. Mann-Whitney *U-*tests are used to compare continuous variables between groups. Our study used logistic regression to explore the associations of BMI and grip strength and the risk of follow-up falls.

The statistical analysis was performed by R version 4.2.1. All estimates were considered statistically significant when *p* < 0.05.

## 3 Results

### 3.1 Participant characteristics

A total of 3,685 participants were included in this study, including 1,813 males and 1,872 females, with an average age of (67.14 ± 6.08) years. Among them, 640 people experienced falls during the baseline in 2011 to the follow-up in 2013, and the incidence of falls in females (21.15%) was much higher than that in males (13.46%). The age, scores of depression, BMI and proportion of people with history of falls in the fall group were higher than those in the non-fall group, whereas the scores of cognition, grip strength and illiteracy rates were lower in the non-fall group. In addition, the degrees of differences between the fall group and the non-fall group vary in terms of marital status, smoking status, alcohol drinking and the number of comorbidities (Table 1).

**Table 1:** Sociodemographic characteristics of participants.

		**Overall (3,685)**	**Non-fall (3,045)**	**Fall (640)**	**U/χ^2^**	***P***
Sex	Male	1,813 (49.2)	1,569 (51.53)	244 (38.12)	37.47	< 0.001
	Female	1,872 (50.8)	1,476 (48.47)	396 (61.88)		
BMI	Normal	2,076 (56.34)	1,701 (55.86)	375 (58.59)	1.80	0.407
	Underweight	682 (18.51)	573 (18.82)	109 (17.03)		
	Overweight and obese	927 (25.16)	771 (25.32)	156 (24.38)		
Group of grip strength	Low	1,066 (28.93)	847 (27.82)	219 (34.22)	10.24	0.001
	High	2,619 (71.07)	2,198 (72.18)	421 (65.78)		
Marital status	Married	2,908 (78.91)	2,436 (80)	472 (73.75)	12.04	< 0.001
	Other	777 (21.09)	609 (20)	168 (26.25)		
Education	Illiterate	1,256 (34.08)	1,015 (33.33)	241 (37.66)	5.98	0.112
	Primary school	1,753 (47.57)	1,460 (47.95)	293 (45.78)		
	Middle school	478 (12.97)	408 (13.4)	70 (10.94)		
	High school and above	198 (5.37)	162 (5.32)	36 (5.62)		
Number of chronic diseases	None	987 (26.78)	843 (27.68)	144 (22.5)	8.49	0.014
	1	1,125 (30.53)	929 (30.51)	196 (30.63)		
	2 and above	1,573 (42.69)	1,273 (41.81)	300 (46.88)		
Smoking status	No	2,153 (58.43)	1,728 (56.75)	425 (66.41)	19.91	< 0.001
	Yes	1,532 (41.57)	1,317 (43.25)	215 (33.59)		
Alcohol drinking	No	899 (24.4)	773 (25.39)	126 (19.69)	9.82	0.007
	Less than once a month	244 (6.62)	203 (6.67)	41 (6.41)		
	More than once a month ^*^	2,542 (68.98)	2,069 (67.95)	473 (73.91)		
History of falls	No	3,028 (82.17)	2,585 (84.89)	443 (69.22)	87.62	< 0.001
	Yes	657 (17.83)	460 (15.11)	197 (30.78)		
Age		66 (9)	66 (8)	66 (9)	913,453.5	0.013
Height		156.3 (12.1)	156.9 (12)	154.3 (11.4)	1,121,901.5	< 0.001
Weight		55.4 (14.4)	55.5 (14.4)	54.85 (14.9)	1,028,593.5	0.027
BMI		22.68 (4.95)	22.65 (4.97)	22.77 (4.91)	943,462	0.206
Scores of cognition		10 (7)	10 (7)	10 (7)	1,025,494	0.036
Scores of depression		8 (8)	7 (8)	9 (9)	850,366	< 0.001
Grip strength		29.5 (13)	30 (12.7)	26.8 (11.65)	1,125,466.5	< 0.001

### 3.2 Multivariate logistic regression analysis

We selected variables with statistically significant differences in univariate analysis and included BMI and grip strength after grouping in the logistic regression model. Calculating the variance inflation factor to evaluate collinearity, there is no evidence to suggest a high correlation between independent variables (Supplementary Table S3). The results of logistic regression analysis are shown in Table 2. Participants with history of falls were 2.281 times more likely to fall than people without history of falls, and the difference was statistically significant (*95 CI:* 1.864–2.786, *P* < 0.001). As age increases, the risk of falls also increases (OR = 1.02, *95 CI*: 1.004–1.036). Participants with high grip strength had lower fall risks compared to those with low grip strength, which was statistically significant in the general population and females. Similarly, the underweight BMI group exhibited a reduced risk of falls compared to the normal BMI group. Higher depression score indicated higher risk of falls, especially in the general population and females, whereas the effect of depression on falls was not statistically significant in males. However, the impact of cognitive conditions on falls in the older adults was insignificant (*P* = 0.137). In addition, amongst females, participants in the separated and divorced/widowed/never married group had higher risk of falls than those who are married (OR = 1.353, 95 *CI*: 1.043–1.750).

**Table 2:** Logistic regression results of risk factors for falls.

		**Overall (3,685)**			**Male (1,813)**			**Female (1,872)**		
		**OR**	**95% CI**	***P***	**OR**	**95% CI**	***P***	**OR**	**95% CI**	***P***
Age		1.02	(1.004–1.036)	0.014	1.005	(0.978–1.031)	0.717	1.029	(1.008–1.05)	0.005
Sex	Male				–	–	–	–	–	–
	Female	**1.479**	**(1.155–1.899)**	**0.002**	–	–	–	–	–	–
Smoking status	No	1			1			1		
	Yes	0.912	(0.719–1.158)	0.452	0.882	(0.646–1.215)	0.437	0.905	(0.615–1.306)	0.604
Alcohol drinking	No	1			1			1		
	Less than once a month	1.131	(0.755–1.666)	0.542	1.096	(0.667–1.751)	0.708	1.341	(0.638–2.748)	0.428
	More than once a month	1.069	(0.84–1.365)	0.592	0.98	(0.726–1.327)	0.898	1.356	(0.877–2.172)	0.186
Marital status	Married	1			1			1		
	Other	1.22	(0.984–1.508)	0.067	0.969	(0.638–1.436)	0.879	1.353	(1.043–1.75)	0.022
Number of chronic diseases	None	1			1			1		
	1	1.116	(0.878–1.42)	0.372	1.111	(0.763–1.622)	0.583	1.082	(0.79–1.487)	0.625
	2 and above	1.138	(0.907–1.433)	0.268	1.352	(0.951–1.937)	0.096	0.996	(0.738–1.35)	0.98
History of falls	No	1			1			1		
	Yes	**2.281**	**(1.864–2.786)**	**< 0.001**	**3.233**	**(2.349–4.426)**	**< 0.001**	**1.823**	**(1.404–2.358)**	**< 0.001**
Group of grip strength	Low	1			1			1		
	High	**0.762**	**(0.63–0.923)**	**0.005**	**0.679**	**(0.503–0.919)**	**0.012**	0.845	(0.659–1.089)	0.19
BMI	Normal	1			1			1		
	Underweight	**0.767**	**(0.595–0.981)**	**0.037**	0.853	(0.584–1.229)	0.401	**0.688**	**(0.486–0.962)**	**0.031**
	Overweight and obese	0.912	(0.732–1.133)	0.408	0.832	(0.561–1.211)	0.346	0.951	(0.727–1.239)	0.71
Scores of cognition		1.017	(0.995–1.041)	0.137	1.014	(0.976–1.053)	0.487	1.019	(0.99–1.049)	0.194
Scores of depression		**1.021**	**(1.006–1.037)**	**0.007**	1.008	(0.981–1.035)	0.557	**1.031**	**(1.011–1.05)**	**0.002**

The bold values highlighted *p* < 0.05.

### 3.3 Subgroup analysis

The results of subgroup analysis are shown in Table 3. Three models were constructed: an unadjusted model including only grip strength (model 1); a model adjusted for socioeconomic factors and lifestyles (model 2) and a model adjusted for health status (model 3). For the general population in underweight group, participants with high grip strength had a 0.56 times higher risk of falls compared to those with low grip strength. After adjusting for covariates, the association between high grip strength in the underweight group and a reduced risk of falls remained statistically significant (OR = 0.56, 95% *CI*: 0.36–0.85), a finding that was similar in females (OR = 0.54, 95% *CI*: 0.29–0.98). In males, the results were relatively different. Males with high grip strength only had lower risk of falls in the normal BMI compared with the low grip strength group. However, the interaction between BMI and grip strength was not significant in all models.

**Table 3:** Multi-model logistic regression results of risk factors for falls by gender.

		**Low grip strength**	**Model 1**		**Model 2**		**Model 3**	
			**High grip strength**	***P*** **for interaction**	**High grip strength**	***P*** **for interaction**	**High grip strength**	***P*** **for interaction**
Overall	Normal	Ref	0.79 (0.62–1.01)	0.36	0.82 (0.64, 1.04)	0.306	0.83 (0.64, 1.07)	0.265
	Underweight	Ref	**0.56 (0.37–0.85)** ^*^		**0.56 (0.37, 0.85)** ^*^		**0.56 (0.36, 0.85)** ^*^	
	Overweight and obese	Ref	0.749 (0.50–1.13)		0.76 (0.50, 1.14)		0.83 (0.54, 1.27)	
Male	Normal	Ref	**0.62 (0.43–0.90)** ^*^	0.848	**0.62 (0.43, 0.89)** ^*^	0.837	**0.63 (0.43, 0.92)**	0.676
	Underweight	Ref	0.63 (0.34–1.14)		0.63 (0.34, 1.14)		0.69 (0.36, 1.32)	
	Overweight and obese	Ref	0.79 (0.38–1.78)		0.79 (0.37, 1.73)		0.95 (0.41, 2.18)	
Female	Normal	Ref	0.97 (0.70–1.35)	0.121	1.03 (0.74, 1.44)	0.136	1.05 (0.75, 1.49)	0.141
	Underweight	Ref	**0.49 (0.27–0.87)** ^*^		**0.54 (0.30, 0.97)** ^*^		**0.54 (0.29, 0.98)** ^*^	
	Overweight and obese	Ref	0.74 (0.46–1.21)		0.73 (0.45, 1.18)		0.77 (0.46, 1.29)	

Model 1: grip strength.

Model 2: Model 1+age+gender+smoking status +alcohol drinking status+ marital status.

Model 3: Model 2+the scores of depression+the scores of cognition + Number of chronic diseases + History of falls.

^*^Indicates *p* < 0.05. The bold values highlighted *p* < 0.05.

## 4 Discussions

In some countries, females are more prone to non-fatal falls, coinciding with a higher incidence of falls in aged females than aged males in this study. Different from males, higher levels of depression increase the risk of falls in aged females (OR = 1.031, 95 *CI*: 1.011–1.020). Women are 70% more likely to suffer from depression in their lifetime, which is caused by a combination of complex social factors (29). Women are more sensitive to the external environment, have a stronger need for social interaction, and are more susceptible to changes in social relationships than men (30). These findings also indicate that poor marital status affects aged female's risk of falls rather than aged male's in our study. Therefore, when taking measures to prevent falls in the older adults population, related departments should focus on the strengthening of social networks of aged female, and considering a course to increase social interaction and reduce social vulnerability in older women, thereby reducing the risk of falls.

Our study found that high grip strength may reduce the risk of falls in the general population and males, which was not significant in females. The influence of muscle strength on falls cannot be ignored. During exercise, the motor cortex drives the movement of the leg muscles through the dermal muscle connection, reducing the risk of falls in the older adults by improving muscle strength as well as the tension and coordination of the musculoskeletal system (31). Males typically have higher muscle mass and different muscle distributions compared to females (32). Even if women have strong grip strength, their muscle mass may still be relatively low, so the relationship between grip strength and falls is not as significant as in males. In addition, females experience a significant decrease in estrogen levels after menopause, which in turn affects the strength and quality of muscles and bones and increases the risk of osteoporosis, resulting in a less pronounced protective effect of grip strength on the risk of falls (33).

In this study, compared with those with normal BMI, the underweight BMI may reduce the risk of falls in the general population and females, and underweight/obese may not increase the risk of falls. This may be because females tend to have higher levels of activity and lower BMI in old age, making them more likely to benefit from it (34). This finding is consistent with most previous studies. Another study based on CHARLS showed a linear correlation between BMI and falls in older adults women, which was also not found in men (35). A review showed that obesity did not increase the risk of falls after menopause in aged females compared with normal-weight aged females (36).

We did not find any interactions between BMI and grip strength, the effects of BMI and grip strength on falls in the older adults are independent of each other. And the impact of BMI on fall risk in the low-grip-strength group is gender-specific in our study. For the aged females with underweight BMI, participants with high grip strength have lower risk of falls than those with low grip strength. In aged males with normal BMI, high grip strength may actually have a protective effect on falls. There may be several reasons for this. For females, underweight may be associated with better physical function and exercise ability, while high grip strength further enhances these advantages, thereby reducing the risk of falls (37). In addition, females with underweight BMI and high grip strength may exhibit better health behaviors, such as active exercise and nutrient intake, thereby reducing the risk of falls (38). In both normal and overweight/obese BMI groups, females may experience less significant protective effects from high grip strength due to higher fat ratios and potential muscle loss. In males, high grip strength under normal BMI can better reflect their muscle strength advantage, thereby reducing the risk of falls. In the underweight and overweight/obese groups, other factors such as malnutrition, overweight, or body imbalance may weaken the protective effect of grip strength (39).

Our study has the following advantages. Firstly, the data of our study are collected from CHARLS, which has a large sample size and authenticity. Thus, our findings can reflect and represent the overall situation of the older adults population in China. Secondly, this research is a prospective longitudinal study, which effectively verifies the causal relationship amongst BMI, grip strength and fall risks in the older adults. Thirdly, based on previous studies and actual situations, our study stratified the data by gender and explored the gender specificity of fall risk. Fourthly, in the subgroup analysis, our study explored the effect of grip strength on fall risk at different grip strength levels, filling the gap in the existing research and enriching the existing theoretical foundation in developing countries.

The present study also has several limitations. Firstly, participants self-reported their falls in our study, which may have some recall bias. However, the questionnaire was collected by well-trained investigators from CHARLS. During the process of filling out the questionnaire, key information was sought from the participants' families for support. For participants who reported injuries due to falls, investigators reviewed their medical records to verify their authenticity, which to some extent reduced recall bias Secondly, the CHARLS data did not contain items that reflected the specific diet and nutrition of the participants and lacked the indicators of the nutritional status of the older adults. The nutritional status of the older adults also affected the actual risk of falls. Although BMI partly reflects the overall health of older people, the impact of micronutrients (such as vitamin D) on falls is not considered (40). In addition, psychotropic drug use can affect the risk of falls in older adults. However, the interference effect of psychotropic drug use has been avoided in the population screening. In the process of selecting participants, we excluded those with brain impairment/intellectual disability. However, the use of psychotropic drugs in other settings cannot be avoided. Secondly, due to the large number of missing values in the physical activity items of the CHARLS questionnaire, we did not include data of physical activity in this study. Finally, the reduced sample size in subgroup analysis may decrease the reliability of the findings.

## 5 Conclusions

This study highlights the potential protective associations of underweight BMI and high grip strength on falls in older adults, noting gender-specific differences in these relationships. While underweight BMI and high grip strength were related to reduced fall risk among older adults, these associations were not statistically significant in older males. Notably, in the underweight BMI group of females, high grip strength is associated with a reduced risk of falls; in the normal BMI group of males, high grip strength has a protective effect on falls. These findings suggest that gender-specific strategies targeting BMI and muscle strength may be effective in reducing fall risks in older adults. However, due to the lack of statistical significance in some findings, these results should be interpreted cautiously, and further research is needed to confirm these associations and understand the underlying mechanisms. Future studies should also consider more comprehensive measures of muscle strength, physical activity, medications used as well as nutritional factors, to better capture the complexity of fall risks in this population. It is worth noting that although lower BMI is associated with a reduced risk of falls in this study, caution is needed when controlling for BMI, as excessively underweight BMI may indicate the onset of sarcopenia (41). Therefore, further research is required to validate the findings of this study.

## Data Availability

The anonymized data collected are available as open data. Full details are available on the website of CHARLS http://charls.pku.edu.cn.

## References

[1] Falls. Available at: https://www.who.int/news-room/fact-sheets/detail/falls (accessed August 3, 2023).

[2] Ye P Er Y Wang H Fang L Li B Ivers R. Burden of falls among people aged 60 years and older in mainland China, 1990-2019: findings from the Global Burden of Disease Study 2019. Lancet Public Health. (2021) 6:e907–18. 10.1016/S2468-2667(21)00231-0 34838197 PMC8646839

[3] Ellmers TJ Delbaere K Kal EC. Frailty, falls and poor functional mobility predict new onset of activity restriction due to concerns about falling in older adults: a prospective 12-month cohort study. Eur Geriatr Med. (2023) 14:345–51. 10.1007/s41999-023-00749-2 36739560 PMC10113287

[4] Pengpid S Peltzer K. Tridirectional association between probable depression, fear of falling and falls among middle-aged and older adults in Thailand. Arch Gerontol Geriatr. (2023) 109:104955. 10.1016/j.archger.2023.104955 36758485

[5] Wu KY Chen DR Chan CC Yeh YP Chen HH. Fear of falling as a mediator in the association between social frailty and health-related quality of life in community-dwelling older adults. BMC Geriatr. (2023) 23:421. 10.1186/s12877-023-04144-1 37430231 PMC10334636

[6] Trevisan C Noale M Mazzochin M Greco GI Imoscopi A Maggi S. Falls may trigger body weight decline in nursing home residents. Nutrition. (2021) 90:111429. 10.1016/j.nut.2021.111429 34481268

[7] Kvelde T McVeigh C Toson B Greenaway M Lord SR Delbaere K. Depressive symptomatology as a risk factor for falls in older people: systematic review and meta-analysis. J Am Geriatr Soc. (2013) 61:694–706. 10.1111/jgs.12209 23617614

[8] Hartley P Forsyth F O'Halloran A Kenny RA Romero-Ortuno R. Eight-year longitudinal falls trajectories and associations with modifiable risk factors: evidence from The Irish Longitudinal Study on Ageing (TILDA). Age Ageing. (2023) 52:afad037. 10.1093/ageing/afad037 36995137 PMC10061938

[9] Stubbs B Binnekade T Eggermont L Sepehry AA Patchay S Schofield P. Pain and the risk for falls in community-dwelling older adults: systematic review and meta-analysis. Arch Phys Med Rehabil. (2014) 95:175–87.e9. 10.1016/j.apmr.2013.08.241 24036161

[10] Chen H Huang L Xiang W Liu Y Xu JW. Association between cognitive frailty and falls among older community dwellers in China: a Chinese longitudinal healthy longevity survey-based study. Front Aging Neurosci. (2023) 14:1048961. 10.3389/fnagi.2022.1048961 36711208 PMC9880264

[11] Muir SW Gopaul K Montero Odasso MM. The role of cognitive impairment in fall risk among older adults: a systematic review and meta-analysis. Age Ageing. (2012) 41:299–308. 10.1093/ageing/afs012 22374645

[12] Holtzer R Friedman R Lipton RB Katz M Xue X Verghese J. The relationship between specific cognitive functions and falls in aging. Neuropsychology. (2007) 21:540–8. 10.1037/0894-4105.21.5.540 17784802 PMC3476056

[13] Allali G Launay CP Blumen HM Callisaya ML De Cock AM Kressig RW. Falls, cognitive impairment, and gait performance: results from the GOOD initiative. J Am Med Dir Assoc. (2017) 18:335–40. 10.1016/j.jamda.2016.10.008 27914848 PMC5366266

[14] Zhang X Huang P Dou Q Wang C Zhang W Yang Y. Falls among older adults with sarcopenia dwelling in nursing home or community: a meta-analysis. Clin Nutr. (2020) 39:33–9. 10.1016/j.clnu.2019.01.002 30665817

[15] Alajlouni DA Bliuc D Tran TS Blank RD Center JR. Muscle strength and physical performance contribute to and improve fracture risk prediction in older people: a narrative review. Bone. (2023) 172:116755. 10.1016/j.bone.2023.116755 37028582

[16] Hong C Choi S Park M Park SM Lee G. Body composition and osteoporotic fracture using anthropometric prediction equations to assess muscle and fat masses - Hong – 2021. J Cachexia Sarcopenia Muscle. (2021) 12:2247–58. 10.1002/jcsm.12850 34706399 PMC8718033

[17] Dowling L McCloskey E Cuthbertson DJ Walsh JS. Dynapenic abdominal obesity as a risk factor for falls. J Frailty Aging. (2023) 12:37–42. 10.14283/jfa.2022.18 36629082

[18] Harvey NC Odén A Orwoll E Lapidus J Kwok T Karlsson MK. Measures of physical performance and muscle strength as predictors of fracture risk independent of FRAX, falls, and aBMD: a meta-analysis of the osteoporotic fractures in men (MrOS) study. J Bone Miner Res. (2018) 33:2150–7. 10.1002/jbmr.3556 30011086 PMC6272117

[19] Leong DP Teo KK Rangarajan S Lopez-Jaramillo P Avezum A Orlandini A. Prognostic value of grip strength: findings from the Prospective Urban Rural Epidemiology (PURE) study. Lancet. (2015) 386:266–73. 10.1016/S0140-6736(14)62000-6 25982160

[20] Mitchell RJ Lord SR Harvey LA Close JCT. Obesity and falls in older people: mediating effects of disease, sedentary behavior, mood, pain and medication use. Arch Gerontol Geriatr. (2015) 60:52–8. 10.1016/j.archger.2014.09.006 25307955

[21] Trevisan C Crippa A Ek S Welmer AK Sergi G Maggi S. Nutritional status, body mass index, and the risk of falls in community-dwelling older adults: a systematic review and meta-analysis. J Am Med Dir Assoc. (2019) 20:569–82.e7. 10.1016/j.jamda.2018.10.027 30554987

[22] Smith L López Sánchez GF Veronese N Soysal P Rahmati M Jacob L. Dynapenic abdominal obesity increases risk for falls among adults aged ≥50 years: a prospective analysis of the Irish Longitudinal Study on Ageing. J Gerontol A Biol Sci Med Sci. (2023) 79:glad104. 10.1093/gerona/glad104 37071490

[23] Yaohui Z John S Gonghuan Y. China Health and Retirement Longitudinal Study (2011 Baseline) [Internet]. Peking University Open Research Data Platform (2015). 10.18170/DVN/IZU8OF

[24] CHARLS. Available at: http://charls.pku.edu.cn/en/ (accessed August 11, 2023).

[25] Chinese Nutrition Society. Appropriate range of body mass index and body weight management guidelines for Chinese oldest old (T/CNSS 021-2023) [J]. Chin J Epidemiol. (2023) 44:1335–7. 10.3760/cma.j.cn112338-20230804-00055 37743262

[26] The Chinese Dietary Guidelines. Available at: http://dg.en.cnsoc.org/ (accessed August 28, 2024).

[27] 国家国民体质监测中心关于发布 《国民体质测定标准（2023年修订）》的通知—-国家体育总局体育科学研究所. Available at: https://www.ciss.cn/tzgg/info/2023/32672.html (accessed August 29, 2024).

[28] Meng Q Wang H Strauss J Langa KM Chen X Wang M. Validation of neuropsychological tests for the China Health and Retirement Longitudinal Study Harmonized Cognitive Assessment Protocol. Int Psychogeriatr. (2019) 31:1709–19. 10.1017/S1041610219000693 31309907 PMC8082093

[29] Kessler RC Berglund P Demler O Jin R Merikangas KR Walters EE. Lifetime prevalence and age-of-onset distributions of DSM-IV disorders in the National Comorbidity Survey Replication. Arch Gen Psychiatry. (2005) 62:593–602. 10.1001/archpsyc.62.6.593 15939837

[30] Choe H Gondo Y Kasuga A Masui Y Nakagawa T Yasumoto S. The relationship between social interaction and anxiety regarding COVID-19 in Japanese Older Adults. Gerontol Geriatr Med. (2023) 9:23337214231175713. 10.1177/23337214231175713 37255654 PMC10225903

[31] Artoni F Fanciullacci C Bertolucci F Panarese A Makeig S Micera S. Unidirectional brain to muscle connectivity reveals motor cortex control of leg muscles during stereotyped walking. Neuroimage. (2017) 159:403–16. 10.1016/j.neuroimage.2017.07.013 28782683 PMC6698582

[32] Patel HP Syddall HE Jameson K Robinson S Denison H Roberts HC. Prevalence of sarcopenia in community-dwelling older people in the UK using the European Working Group on Sarcopenia in Older People (EWGSOP) definition: findings from the Hertfordshire Cohort Study (HCS). Age Ageing. (2013) 42:378–84. 10.1093/ageing/afs197 23384705 PMC3633365

[33] Buckinx F Landi F Cesari M Fielding RA Visser M Engelke K. Pitfalls in the measurement of muscle mass: a need for a reference standard. J Cachexia Sarcop Muscle. (2018) 9:269–78. 10.1002/jcsm.12268 29349935 PMC5879987

[34] Hernandes NA Probst VS Da Silva RA Januário RSB Pitta F Teixeira DC. Physical activity in daily life in physically independent elderly participating in community-based exercise program. Braz J Phys Ther. (2013) 17:57–63. 10.1590/S1413-35552012005000055 23117651

[35] Zhao X Yu J Hu F Chen S Liu N. Association of body mass index and waist circumference with falls in Chinese older adults. Geriatr Nursing. (2022) 44:245–50. 10.1016/j.gerinurse.2022.02.020 35248838

[36] Sotiriadi-Vlachou S. Obesity and its relationship with falls, fracture site and bone mineral density in postmenopausal women. J Frailty Sarcopenia Falls. (2017) 2:28–32. 10.22540/JFSF-02-028 32300680 PMC7155376

[37] Ylitalo KR Karvonen-Gutierrez CA Sternfeld B Pettee Gabriel K. Association of physical activity and physical functioning phenotypes with fall risk among women. J Aging Health. (2021) 33:409–17. 10.1177/0898264320988405 33517822 PMC8356562

[38] Aragon AA Schoenfeld BJ Wildman R Kleiner S VanDusseldorp T Taylor L. International society of sports nutrition position stand: diets and body composition. J Int Soc Sports Nutr. (2017) 14:16. 10.1186/s12970-017-0174-y 28630601 PMC5470183

[39] Clynes MA Edwards MH Buehring B Dennison EM Binkley N Cooper C. Definitions of sarcopenia: associations with previous falls and fracture in a population sample. Calcif Tissue Int. (2015) 97:445–52. 10.1007/s00223-015-0044-z 26223791 PMC4601152

[40] Chevalley T Brandi ML Cashman KD Cavalier E Harvey NC Maggi S. Role of vitamin D supplementation in the management of musculoskeletal diseases: update from an European Society of Clinical and Economical Aspects of Osteoporosis, Osteoarthritis and Musculoskeletal Diseases (ESCEO) working group. Aging Clin Exp Res. (2022) 34:2603–23. 10.1007/s40520-022-02279-6 36287325 PMC9607746

[41] Curtis M Swan L Fox R Warters A O'Sullivan M. Associations between body mass index and probable sarcopenia in community-dwelling older adults. Nutrients. (2023) 15:1505. 10.3390/nu15061505 36986233 PMC10059806

