# Supplementary material for: Effects of BMI and grip strength on older adults' falls—A longitudinal study based on CHARLS
Source: Front Public Health. 2024 Dec 4;12:1415360. doi: 10.3389/fpubh.2024.1415360 (PMC11652278; doi:10.3389/fpubh.2024.1415360)
Supplement: Supplementary file 1 [file Table_1.docx]

Table S1 BMI Cut-offs in different ages

| Age | Level | Cut-offs |
| --- | --- | --- |
| 60~64 | underweight | ~18.4 |
|  | Normal | 18.5~23.9 |
|  | Overweight and obese | 23.9~ |
| 65-80 | underweight | ~19.9 |
|  | Normal | 20~26.8 |
|  | Overweight and obese | 26.9~ |
| 80~ | underweight | ~21.9 |
|  | Normal | 22~26.8 |
|  | Overweight and obese | 26.9~ |

Table S2 Grip strength Cut-offs in different ages and genders

|  | Age | Cut-offs |
| --- | --- | --- |
| Male | 60~64 | 33.9 |
|  | 65~69 | 32.1 |
|  | 70~74 | 29.3 |
|  | 75~ | 26.7 |
| Female | 60~64 | 21.7 |
|  | 65~69 | 20.8 |
|  | 70~74 | 19.5 |
|  | 75~ | 18.8 |

Table S3 Variance Inflation Factors in Logistic Regression Models

|  | Overall | Male | Female | Df |
| --- | --- | --- | --- | --- |
| Age | 1.303794714 | 1.221459433 | 1.378942272 | 1 |
| Sex | 1.967769425 |  |  | 1 |
| Smoking status | 1.718446299 | 1.053953216 | 1.017548292 | 1 |
| Alcohol Drinking | 1.277358281 | 1.073780205 | 1.02878322 | 2 |
| Marital Status | 1.120417127 | 1.046491728 | 1.139212808 | 1 |
| Number of Chronic Diseases | 1.077843729 | 1.079837594 | 1.082383674 | 2 |
| History of Falls | 1.037108692 | 1.030121474 | 1.038537935 | 1 |
| Group of Grip Strength | 1.062889154 | 1.090431784 | 1.062942827 | 1 |
| BMI | 1.236953146 | 1.237233807 | 1.211173288 | 2 |
| Scores of Cognition | 1.280112951 | 1.170813254 | 1.208961447 | 1 |
| Scores of Depression | 1.157032766 | 1.135045464 | 1.146806159 | 1 |

Table S4 Hosmer and Lemeshow goodness of fit test in different models

|  | X-squared | df | P |
| --- | --- | --- | --- |
| Overall | 7.4886 | 8 | 0.4849 |
| Overall_Normal_BMI | 13.62 | 8 | 0.09222 |
| Overall_Low_BMI | 9.111 | 8 | 0.333 |
| Overall_High_BMI | 2.2989 | 8 | 0.9705 |
| Male | 5.0846 | 8 | 0.7485 |
| Male_Normal_BMI | 6.4805 | 8 | 0.5936 |
| Male_Low_BMI | 10.455 | 8 | 0.2345 |
| Male_High_BMI | 9.8699 | 8 | 0.2743 |
| Female | 4.2134 | 8 | 0.8374 |
| Female_Normal_BMI | 4.7894 | 8 | 0.7798 |
| Female_Low_BMI | 3.5829 | 8 | 0.8927 |
| Female_High_BMI | 7.9444 | 8 | 0.4389 |
